# Genome-Wide Association Study as an Efficacious Approach to Discover Candidate Genes Associated with Body Linear Type Traits in Dairy Cattle

**DOI:** 10.3390/ani14152181

**Published:** 2024-07-26

**Authors:** Mingxue Long, Bo Wang, Zhangping Yang, Xubin Lu

**Affiliations:** 1 College of Animal Science and Technology, Yangzhou University, Yangzhou 225009, China; mz120231552@stu.yzu.edu.cn (M.L.); yzp@yzu.edu.cn (Z.Y.); 2 College of Food Science and Engineering, Yangzhou University, Yangzhou 225009, China; wb@yzu.edu.cn

**Keywords:** body linear type traits, GWAS, genetic markers, genomic selection, dairy cows

## Abstract

**Simple Summary:**

This study synthesizes reports on body conformation traits in dairy cows, emphasizing their importance in dairy farming. It elucidates the potential role of these traits in enhancing dairy cattle breeding programs by summarizing their genetic parameters and impact on size, conformation, production, health, and fitness. The review underscores the use of genome-wide association studies (GWAS), a powerful genetic tool that identifies specific genes and genomic regions influencing body conformation. By integrating global research on these genetic markers, the study offers valuable insights for advancing the selection and breeding of dairy cows, ultimately benefiting farmers and the dairy industry through more informed breeding strategies and improved herd quality.

**Abstract:**

Body shape traits are very important and play a crucial role in the economic development of dairy farming. By improving the accuracy of selection for body size traits, we can enhance economic returns across the dairy industry and on farms, contributing to the future profitability of the dairy sector. Registered body conformation traits are reliable and cost-effective tools for use in national cattle breeding selection programs. These traits are significantly related to the production, longevity, mobility, health, fertility, and environmental adaptation of dairy cows. Therefore, they can be considered indirect indicators of economically important traits in dairy cows. Utilizing efficacious genetic methods, such as genome-wide association studies (GWASs), allows for a deeper understanding of the genetic architecture of complex traits through the identification and application of genetic markers. In the current review, we summarize information on candidate genes and genomic regions associated with body conformation traits in dairy cattle worldwide. The manuscript also reviews the importance of body conformation, the relationship between body conformation traits and other traits, heritability, influencing factors, and the genetics of body conformation traits. The information on candidate genes related to body conformation traits provided in this review may be helpful in selecting potential genetic markers for the genetic improvement of body conformation traits in dairy cattle.

## 1. Introduction

Body linear type traits in dairy cattle are widely recognized as one of the most crucial economic traits. These traits not only play a pivotal role in dairy cow selection but are also integral to evaluating other production-related traits such as milk production, reproduction, mobility, longevity, and feed intake [1]. Serving as indirect predictors, these traits facilitate the selection process for improving productivity and health in dairy herds [2]. Research estimates that dairy cows have a natural lifespan of about 20 years. However, they are often culled much earlier, primarily due to low milk production or health issues. Interestingly, while cows with lower milk production are culled at a higher rate, high-producing cows are not without their risks, facing significant health challenges that can also lead to culling [3]. These dynamic underscores the importance of balancing productivity with health and longevity to maximize corporate profits and sustainability. In this light, the dairy industry, facing expanding pastures and rising labor costs, increasingly prioritizes key adaptive characteristics such as health, fertility, and survival. Longer-lived cows not only produce more milk over their lifetimes but also reduce replacement costs and disease risk, enhancing the economic and environmental sustainability of farms. In China, the culling of most Holstein dairy cows before their third calving, due to reproductive issues, limb and hoof diseases, udder health concerns, and kidney disease, highlights the importance of these traits [4]. Monitoring body linear type traits from an early stage, particularly during the first lactation, provides valuable indirect indicators of a cow’s potential productive life [5]. Studies have demonstrated a strong correlation between specific body linear type traits’ aspects—like body size, rump structure, leg and foot configuration, and udder system—and animal longevity [6]. For instance, cows with straight legs and steeper foot angles tend to have longer productive lives, while a smaller body size is associated with increased lifespan. Conversely, animals exhibiting narrow chests, short rump heights, severely sickled legs, and low foot angles face a higher risk of culling [7].

Understanding the genetics underlying body linear type traits is as crucial as understanding production traits from an economic perspective. With the goal of enhancing the profitability of the dairy industry, selecting for body linear type traits emerges as a feasible strategy. Recent advances in dairy cattle genetic improvement programs worldwide have leaned towards selecting traits related to body linear type traits, driven by their growing economic significance. The success of these programs hinges on the identification of genetic markers, quantitative trait loci (QTL), and genes associated with body conformation.

The advent of high-density array technologies has paved the way for identifying novel genetic markers critical to economically important traits in cattle, including single nucleotide polymorphisms (SNPs) [8]. Given their stability, heritability, and abundance across species genomes, SNPs have been widely utilized in GWASs to uncover the genetic architecture of crucial economic traits [9,10]. A GWAS, an established method in the animal production industry, leverages phenotype and genotype data to identify causal genetic markers for various economic traits using appropriate statistical models [11,12]. Extensive research across different breeds and regions has aimed to identify genetic markers for body linear type traits in cattle, underscoring the global effort to enhance dairy cattle productivity and health through genetic insights.

This review aims to comprehensively evaluate the effectiveness of GWASs in identifying key candidate genes and genomic regions related to body conformation traits in dairy cows. It explores the significance of these traits, their relationships with other traits, heritability, influencing factors, and underlying genetics, and provides potential genetic markers for improving body conformation traits, thereby promoting the economic and sustainable development of the dairy industry.

## 2. The Importance of Body Linear Type Traits

Selecting the correct cow body linear characteristics is critical to improving the productivity and health of the dairy herd. This section delves into the utility of body linear type traits in dairy cattle, examining their role as indicators of various functional efficiencies, including feed efficiency, longevity, milk production, reproductive health, and overall animal welfare [13,14,15,16]. By capturing the diverse applications of these traits in genetic selection and herd management, we illuminate how they contribute to the sustainable optimization of dairy operations. Some of the results on the effects of body shape traits on dairy cow production are summarized in Table S1.

### 2.1. Body Linear Type Traits as Feed Efficiency Indicators

Recording body linear type traits, typically conducted during a cow’s first lactation [17], is an essential component of dairy recording programs and serves as a viable strategy for assessing feed efficiency. The dairy industry faces challenges and high costs in measuring individual feed intakes, which restrict the scope of genetic studies on feed intake and utilization [18]. Nonetheless, body linear type traits offer a promising alternative, providing insights into feed intake and utilization through indirect selection based on traits that have moderate to strong correlations with individual feed consumption [18]. For instance, cows with optimal body condition and udder traits are often more efficient at utilizing feed, supporting higher milk production without excessive feed intake [19]. Traits such as body size, stature, body condition, and udder conformation are significantly related to body weight and play a vital role in regulating feed efficiency and energy balance, which is essential for the financial and biological efficiency of dairy production [20]. Additionally, traits indicating dairy angularity and muscularity may signal issues stemming from a negative energy balance or specific metabolic reserves. Manafiazar’s research underlines the potential of combining conformation traits—stature, dairy strength, chest width, rear udder attachment, and pin width—which exhibit moderate to strong genetic correlations with feed efficiency traits, particularly residual feed intake [18]. Leveraging these indicators allows farmers and breeders to identify cows that not only produce milk more efficiently but also contribute to more sustainable dairy farming practices by minimizing waste and enhancing herd efficiency.

### 2.2. Body Linear Type Traits and Longevity

Cow longevity is critical to economic performance and herd sustainability [21], encompassing a combination of characteristics that influence a cow’s ability to remain in the herd over the long term [22]. Selection for longevity is challenged by its low heritability and myriad influencing factors, leading to a focus on linear body traits as indirect but valid indicators [5]. Dairy professionals worldwide are working to extend cattle lifespan by optimally scoring conformation characteristics that correlate with longevity [22], including chest width, body height, rump width, and specific mammary system traits such as teat length and udder depth, all of which demonstrate a strong genetic correlation [23]. These traits reflect not only an animal’s current health and productivity but also predict long-term viability, help mitigate common health problems, and enhance reproductive performance. Selection practices favoring desirable linear body traits improve the genetic quality of the herd, producing cows with higher milk production, better recovery from stress and disease, and an extended productive life [24]. This holistic approach reduces the need for frequent replacements and enhances animal welfare, underscoring the intricate link between linear body characteristics and cow longevity [5].

### 2.3. Predicting Milk Production Using Body Linear Type Features

The primary goal of cattle breeding programs is to increase milk production. Utilizing body linear type characteristics to predict milk production offers a practical method for the dairy farming industry. Accurate measurement and prediction of milk production are crucial for the economic viability of the dairy industry [25]. Measuring body size linear traits early, before calculating the estimated breeding value (EBV) for milk production, allows these traits to serve as important indicators of milk production EBV. If these predictors do not adversely affect cow profitability, mating decisions can incorporate conformational features [26]. Studies have shown genetic correlations between body linear type traits and first lactation milk production, ranging from 0.48 to 0.54, highlighting the importance of understanding these relationships for improving milk production and body linear traits [27]. The relationship between milk production traits and body weight is complex and influenced by the size of the cow and linear body traits [28]. Studies have highlighted a negative genetic relationship between body weight at first calving and milk, fat, and protein production [29]. Genetically heavier cattle tend to show lower post-calving production but higher conception rates. Conformational features of the mammary system, such as teat position, udder attachment, and udder mass, are critical to maximizing milk production and its composition, promoting efficient milking, and ensuring product longevity [30]. Cows with higher milk production typically exhibit taller hind udders, shallower udder depth, and more pronounced central udder ligaments than lower-producing cows. Focusing selection on mammary system scoring may improve not only milk production but also milking comfort, teat health, and udder health, thus providing an integrated approach to enhancing dairy cow productivity through genetic and conformational considerations [31]. Therefore, the analysis of linear characteristics of body size provides a valuable tool for predicting and improving milk production, ensuring the economic viability, and promoting the environmental sustainability of dairy farming. 

### 2.4. The Effect of Body Linear Type Traits on Reproduction

In dairy cows, the pelvic region and loin structures serve as crucial links between the lumbar region, abdomen, and the extremities, including the feet, legs, and the mammary system [32]. The structural integrity of these areas is crucial for a cow’s productive lifespan, as weaknesses can significantly impact fertility and mobility. Specifically, the design of the pelvic structure influences calving ease and calf survival—key factors that affect the profitability of dairy farming due to the economic losses associated with them [33]. Dystocia, or difficult calving, adversely affects several key performance indicators, including the number of services per conception, the duration of open days, cow mortality, and the yields of milk, fat, and protein during lactation [34]. Optimal pelvic configurations, which are characterized by a wide and appropriately sloped rump, facilitate smoother calf delivery and efficient drainage of post-calving fluids, thereby reducing the risks of fertility issues and metritis. The ideal rump phenotype for facilitating calving includes pin bones that are slightly lower than the hook bones, coupled with a long, wide rump that possesses a distinctive shape and a vulva that presents almost vertically from the side. Conversely, elevated pin bones lead to an undesirable inclination of the vaginal canal, increasing the risk of infections due to inefficient drainage. These anatomical predispositions, particularly in cows with narrow rumps and elevated pin bones, are genetically linked to longer calving intervals and increased calving difficulties, and also predispose them to retained placentas [35,36].

Research has elucidated the correlation between specific body linear type traits, particularly those associated with milk yield, and fertility parameters like the calving interval [37] Findings by Melendez et al. and Wall et al. indicate that poor conformation of legs and feet negatively impacts calving intervals [38]. An unfavorable genetic correlation with rump angle suggests that animals with elevated pin bones experience prolonged intervals between calvings. These insights highlight the significance of incorporating body linear type traits into breeding strategies, emphasizing their influence not only on milk production but also on reproductive efficiency and overall herd health, thereby fostering more sustainable dairy farming practices. 

### 2.5. Movement and Health of Feet and Legs

An increasing number of animal breeders and breeding programs are focusing on structural features related to the feet and legs of animals to improve productivity and profitability [39]. For dairy cows, foot and leg problems are the third most common reason for slaughter, following reproductive issues and udder health problems [14]. Efficient production, reproduction, health, and welfare are all directly related to adequate exercise [40]. Several studies have measured structural traits of feet and legs in dairy cows using various indicator traits. These include the posterior view of the hind leg, the lateral view of the hind leg, foot angle, and bone mass [1]. Genetic studies have found moderate to high correlations between the architectural characteristics of the feet and legs and the prevalence of health problems such as lameness and claw disease in these areas [41]. This suggests that these characteristics may serve as indirect selection criteria for preventing foot and leg health problems.

### 2.6. Health of Mammary System in Dairy Cattle

Mammary system traits play an important economic role in dairy cattle. Infection of the mammary gland is the main cause of mastitis in dairy cows. Udder tissue serves as the primary natural defense against the entry of pathogens through the teat ducts, counteracting negative effects on the animal’s udder health. Several studies have shown that intramammary infections pose a significant threat to the dairy industry. Udder health diseases can cause severe economic losses and significantly impact dairy cow welfare and productivity [42]. When aiming for type improvement, mammary gland traits should receive more selective emphasis, as these traits are directly related to udder health and, consequently, to the economic impact on the dairy herd [43]. Genetic correlations between fitness and conformational traits generally range from moderately negative to moderately positive (−0.32 to 0.37), suggesting that selection for improved udder conformation can reduce somatic cell counts and the incidence of clinical mastitis [44]. Selecting for conformational features can enhance overall health; notably, animals with superior udder architecture are less susceptible to mastitis, experience less trauma, and have increased milk production [45].

### 2.7. Effects of Environmental Factors on Body Size Linear Traits

Extreme environmental factors can adversely affect dairy cow production. Therefore, it is essential to monitor closely the environmental impact on economically important traits. Understanding body size linear traits provides a genetic basis for inter-individual variation and is fundamental in animal production areas such as reproduction, health, and other economically relevant traits. This knowledge is crucial for identifying mechanisms of environmental adaptation in livestock [46]. According to a publication on cattle genetic resources, Tibetan cattle residing on the Qinghai–Tibet Plateau are among the shortest of the native Chinese breeds [47]. This observation underscores the significant role of body size in adapting to harsh environments. Lower temperatures may result in increased birth weights in calves, as the cow’s physiological response to cold includes enhancing blood flow to vital internal organs, including the uterus. This adaptation maintains the calf’s body temperature and nutrient supply, optimizing heat conservation and nutrient uptake. Research has shown that factors like high altitudes, plains, and forested mountains significantly correlate with an increased weaning weight and yearling weight in cattle [48]. Consequently, it is imperative to adjust performance evaluations based on desired traits and to customize breeding programs to suit the specific environmental conditions where the animals are raised. 

### 2.8. Heritability of Linear Body Traits 

Linear body traits are intrinsically linked to milk production, reproduction, lameness, mastitis, and herd longevity in dairy cows [49]. Accurately estimating genetic parameters for these economically significant traits is vital for the development and execution of effective breeding programs. Such estimations are fundamental for genomic research, including GWASs and genomic selection (GS). Many countries incorporate linear body traits into their dairy cattle breeding strategies; thus, precise heritability estimates are crucial for forecasting expected selection responses and calculating breeding values [50]. Research shows that the heritability of linear body traits generally varies from low to moderate. For instance, heritability estimates for these traits in the Italian Jersey breed are low, whereas height displays moderate heritability at 0.32. Heritability for other traits like hip width and hip angle spans from 0.06 to 0.14. Traits related to hooves and legs exhibit the lowest heritability, with figures such as 0.04 for hind leg views and locomotion, and 0.07 for foot angle [51]. A study by Olasege on Chinese Holstein cattle presented heritability estimates ranging from low (0.04 for hoof and leg traits) to moderate (0.23 for body mass), assessed using a composite index [52]. Table 1, Table 2, Table 3 and Table 4 summarize the heritability of linear body traits in dairy cows, including body size traits (Table 1), feet and leg conformation traits (Table 2), rump traits (Table 3), and mammary system traits (Table 4).

## 3. Importance of GWASs in Dairy Cattle Breeding Programs

Genomics is a pivotal method used to achieve significant genetic improvements in animal breeding. This branch of science delves into genomes to identify genes in livestock associated with economically significant traits [61]. It involves the genotyping of several hundred thousand DNA markers dispersed across the genome [62]. A variety of powerful statistical genetic tools are available for identifying alleles that govern target traits [63]. GWASs are among these methodologies, successfully deployed to pinpoint candidate genes for key traits in dairy cattle. These studies explore the relationship between specific molecular markers, such as SNPs or genomic regions, and the traits of interest [64]. GWASs have revealed profound genotype–phenotype correlations and the genetic structure of complex traits across numerous animal species, including cattle [64], buffalo [65], sheep [66], goat [67], chicken [68], and others. GWASs’ contributions have advanced gene editing and functional biology, enhanced the efficiency of genomic selection, and provided extensive insights into the genetic architecture of complex traits and disease susceptibilities [69]. 

### 3.1. How to Conduct a GWAS

**Figure 1:**
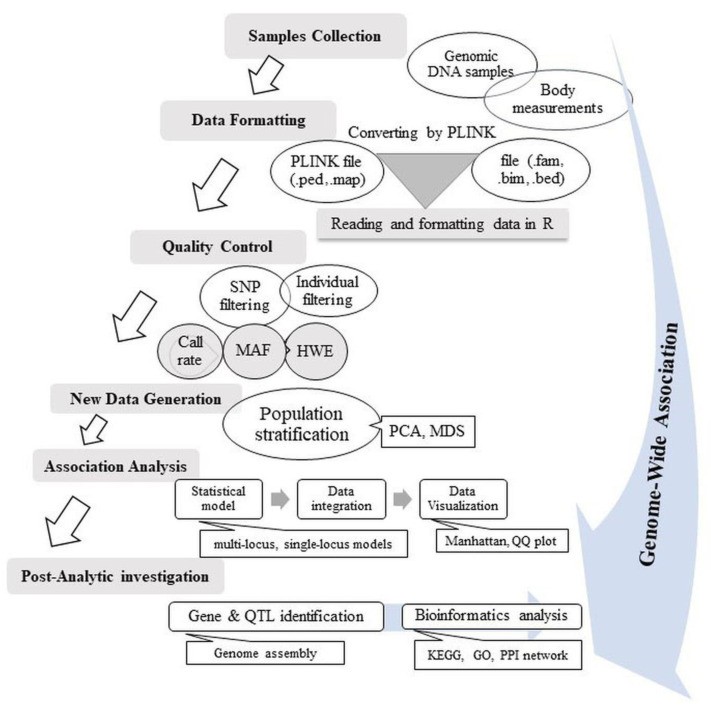
Essential steps for performing a successful GWAS experiment.

Several steps are required to conduct a GWAS experiment successfully, as illustrated in Figure 1. The first step involves selecting the study population, considering a size of appropriate individuals to minimize the negative impact on study power. The population size is pivotal for the success of a GWAS, as it is regarded as a limiting factor for the statistical power of the analysis. Ideally, the sample size should be increased as much as possible to ensure sufficient statistical power to detect associations with significant effects, acceptable frequencies within the population, and to address rare variations [70]. A GWAS’s statistical power is defined as the probability of correctly rejecting a false null hypothesis. The effective sample size represents the minimum number of samples required to achieve adequate statistical power. In large-scale association studies, maintaining 80% statistical power is crucial to minimize false-negative findings and to determine a cost-effective sample size. Typically, in livestock genetics research, a cohort of at least 1000 individuals is considered sufficient, achieving about 80% GWAS statistical power [71]. In contrast, a smaller sample size compromises the reliability of the study by increasing the rate of false negatives and reducing the detection capability of true associations. While GWASs can be conducted with sample sizes between 100 and 500 individuals, smaller cohorts may affect the robustness of the results [70]. However, it is important to note that proper selection of animals, such as those with different genetic backgrounds, can significantly reduce the required sample size while maintaining a low false discovery rate [32,72]. Specifically, crossbred populations, especially those from F2 breeding designs, are preferable due to their higher linkage disequilibrium (LD). This increased LD enhances the power of GWASs, thereby reducing the sample size needed for robust results [73,74]. 

The second step, accurate phenotyping, is crucial for detecting genotype–phenotype associations. All genotyped individuals should be phenotyped for specific traits aligned with the study objectives [69]. To ensure analytical accuracy, deviations from normal distribution assumptions, including outlier removal, must be addressed [71]. Boxplots provide an efficient means to identify and exclude extreme outliers, thereby maintaining the integrity of the analysis [63]. In this process, the heritability of a trait can be estimated from raw phenotypes and relationships derived from pedigrees or genotypes, while potentially accounting for factors such as environmental variation, genetic dominance, or epistatic interactions. High heritability suggests that the trait is predominantly determined by genetic factors, aiding in the identification of association signals [63]. 

In the third step, the same set of phenotyped individuals must be genotyped using DNA molecular markers. Post-genotyping, a quality control step is conducted to minimize the risk of detecting false positive or false negative associations. A false positive association (Type I error) occurs when an association is identified between a SNP and the trait under study that does not actually exist. Conversely, a false negative association (Type II error) arises when a SNP that affects the trait of interest is not identified as being associated with the trait in the study [75]. Before performing quality control on a ‘per-marker’ basis, it is advisable to first perform quality control at the individual level to preserve as many markers as possible. This method ensures that markers are not mistakenly removed due to poor genotyping in a subset of individuals. Nevertheless, there is a risk of erroneously excluding individuals due to poorly genotyped subsets of markers. Per-individual quality control in GWASs involves checking for discrepancies in sex information, presence of missing genotypes or inadequate heterozygosity rates, identifying duplicate or related individuals, and detecting individuals with divergent ancestry [76].

There are at least three essential steps involved in GWAS data quality control for each marker: I—Identification of SNPs with a high number of missing genotypes. This step involves discarding SNPs with a low genotype call rate. The call rate is defined as the percentage of individuals in the study for whom the data on a specific SNP is available; II—Identification and removal of markers with a very low minor allele frequency (MAF). MAF quantifies the variation of a particular SNP across the study population, and a very low MAF indicates inadequate statistical power to detect significant associations with the traits of interest; III—Removal of SNPs that show significant deviation (*p* values ranging between 10^−5^ and 10^−7^) from the Hardy–Weinberg equilibrium (HWE). Deviations from the HWE suggest potential population substructure or genotyping errors, and such deviations are assessed using the Chi-square test; IV—Population Stratification in GWASs, in which genetic markers are identified that contribute to the development and progression of specific traits. 

In the fourth step, it is essential to evaluate the population structure before proceeding with a GWAS [77]. A GWAS typically focuses on the statistical relationships between phenotypic traits and genetic markers, such as SNPs. However, GWAS results can be confounded by spurious associations if sample structures are not correctly interpreted [78]. Sample structure, which includes family structure and cryptic relatedness, refers to the relatedness between individuals within a population cohort. Genetic relatedness within a cohort can prevent standard association studies from accurately identifying causal markers, leading to false positive results [79]. Moreover, there is a greater degree of genetic relationship among individuals sharing ancestry than among those from different ancestries. Cryptic relatedness describes relationships between closely related individuals whose shared ancestry remains unknown to researchers [80]. Indeed, population structure poses a significant source of confounding in a GWAS [81]. Consequently, it is essential to characterize population structure effectively to avoid false positives (Type I errors) or false negatives (Type II errors) in SNP-trait associations. To address population stratification in a GWAS, appropriate models must be applied to demonstrate that corrections have been made effectively. The genomic inflation factor (λ) assesses the effectiveness of models in controlling for population structure [82]. Ideally, λ should equal 1, indicating no stratification. When λ exceeds 1, GWAS results can be confounded by factors such as stratification, family structure, or cryptic relatedness. Several methods, including principal component analysis (PCA) and multidimensional scaling (MDS) [83], have been developed to correct for population stratification. PCA identifies the principal components that represent the major axes of genetic variation, effectively summarizing population structure, while MDS encompasses a broader range of techniques, including PCA, and is used to detect underlying dimensions that explain observed genetic distances.

The fifth step involves detecting the LD across the genome. The extent of LD across the genome significantly influences the efficacy of a GWAS, and this extent varies among different breeds and population groups [84]. LD between molecular markers indicates the correlation between the genotypes of two markers or the degree of non-random association between their alleles. Measuring LD is vital for estimating the distance between loci and determining the necessary number of markers to adequately cover the genome [85]. A high LD suggests fewer markers are required. Moreover, understanding the allele phase relationships between markers and QTLs is essential to ascertain the extent of LD across populations. LD data can also shed light on population history, selective breeding practices, and genetic mutations within specific genomic areas. The r-squared statistic (r^2^) is typically employed to predict LD extent. Long-range LD can lead to false associations; hence, it is crucial to compute LD at the start of the association analysis to reduce such errors. Long-range LD blocks are more prevalent in livestock, particularly in dairy cattle, compared to humans. This prevalence is due to the selective breeding involving a limited number of sires, which results in a smaller effective population size [86]. LD decay, the rate at which LD diminishes with genetic or physical distance, significantly impacts the number of markers required for an effective GWAS. Rapid LD decay necessitates a large number of markers for comprehensive genome association analyses. LD decay can be visualized through scatter plots and heatmap plots, which plot r^2^ values against genetic or physical distances among SNP pairs throughout a genome, a chromosome, or within specific genomic areas like QTLs.

The sixth step entails selecting the most suitable statistical model. Common methods include the general linear model (GLM), logistic mixed model (LMM), mixed linear model (MLM), and compressed mixed linear model (CMLM), which are primarily single-locus analyses using fixed SNP effect mixed linear models. Given the large number of markers, a Bonferroni correction for multiple testing is typically applied, though it may be excessively stringent [87]. To address this, multi-locus models like the Fixed and Random Models Cyclic Probability Unification (FarmCPU) and the multi-locus mixed linear model (MLMM) are proposed, offering advantages in estimating three variance components, enhancing QTLs detection, and treating SNP effects as random [78]. After selecting the GWAS model, relevant software can be used to analyze phenotypic and genotypic data to identify alleles associated with specific traits, and a list of available software is provided in Section 3.3 of this manuscript. It is important to recognize that genotype–environment interactions can influence the effectiveness of association analysis. These interactions, which describe how different genotypes respond under various environmental conditions, significantly affect animal performance and introduce variability based on environmental context. In a GWAS, understanding these interactions is essential for elucidating the genetic architecture of complex traits and for accurately identifying associations between phenotypes and genotypes [88]. Additionally, incorporating environmental effects into the analysis significantly improves the precision of QTL detection [89].

The final step is to display GWAS results and conduct QTLs mining detection. The significance of markers is often expressed using a threshold of −log10 *p* value, usually set by the false discovery rate (FDR) or Bonferroni correction [90,91]. These methods, designed for multiple comparisons, allow for significance testing across hundreds of thousands to millions of markers. GWAS results are typically presented in Manhattan plots and quantile–quantile (QQ) plots, supplemented by tables listing significant SNPs, minor allele frequency, sample size, proportion of phenotypic variance explained by the markers (R^2^), and adjusted *p* values (based on the significance threshold determined by either Bonferroni correction or FDR).

### 3.2. GWAS SNP Chips

Since 2006, high-density single nucleotide polymorphism (SNP) panels have been utilized in livestock and plant genomics, significantly enhancing genetic selection in dairy cattle [81]. Commercial SNP arrays are diverse, developed by leading companies such as Illumina, Neogen (formerly Geneseek), and Affymetrix, each offering platforms that genotype SNPs at varying densities [82]. These range from the Golden Gate Bovine 3 K, containing 2900 SNPs, to the comprehensive Bovine HD, which includes 777,962 SNPs. The success of a GWAS hinges on the density of these SNP arrays [71]. However, the prohibitive cost of high-density arrays can restrict the sequencing scope for extensive animal cohorts. To circumvent this, genotype imputation is commonly employed to bridge low-density SNP arrays to high-density versions or even whole genome sequencing, thus optimizing costs [92]. This technique not only broadens the array of detectable variants for association testing, including those of low frequency and rarity, but also has proven accuracy in transitioning from lower to higher density SNP arrays or full genomic sequencing, as validated by numerous studies [87,93].

### 3.3. Genomic Databases and Software for GWAS Analysis

A variety of free software programs are available for GWAS analysis, commonly including PLINK (http://pngu.mgh.harvard.edu/purcell/plink, accessed on 1 May 2024 [94]), R GenABEL (https://cran.r-project.org/src/contrib/Archive/GenABEL, accessed on 1 May 2024 [95]), and GenAMap (http://cogito-b.ml.cmu.edu/genamap, accessed on 1 May 2024 [96]). These tools are essential for population stratification, quality control, LD, and structured association mapping. Additionally, GEMMA (Genome-wide Efficient Mixed Model Association, http://www.xzlab.org/software.html, accessed on 1 May 2024) is utilized for population stratification, analysis of identical by descent (IBD), estimation of chip heritability, and association mapping. BLUPF90 (http://nce.ads.uga.edu/wiki/doku.php?id=documentation, accessed on 1 May 2024) facilitates GWASs, data conditioning, variance estimation through various methods, and enhancement of breeding value accuracy using SNP data. Publicly accessible web databases hosting genomic study data include NCBI (National Center for Biotechnology Information Gene, https://www.ncbi.nlm.nih.gov, accessed on 1 May 2024), Animal QTLdb (Animal Quantitative Trait Loci Database, https://www.animalgenome.org/cgi-bin/QTLdb/index, accessed on 1 May 2024), NAGRP (National Animal Genome Research Program, https://www.animalgenome.org, accessed on 1 May 2024), EMBL-EBI (European Molecular Biology Laboratory-European Bioinformatics Institute, https://www.ebi.ac.uk, accessed on 1st May 2024), DDBJ (DNA Data Bank of Japan), UCSC (University of California Santa Cruz Genome Browser, https://genome.ucsc.edu, accessed on 1 May 2024), RefSeq (Reference Sequence Database, same UCSC link), and VEGA (Vertebrate Genome Annotation, http://vega.archive.ensembl.org/index.html, accessed on 1 May 2024). Some databases are specifically tailored to livestock genomics, while others provide broad access for research across various organisms. 

### 3.4. Post GWAS

Exploring the genetic architecture of traits necessitates conducting post-GWAS analyses to understand the interactions among genomic regions and to identify SNPs and/or regions associated with target traits. These analyses include functional evaluations such as KEGG pathways and gene ontology terms, and the construction of biological networks. While gene network analyses have been applied in research across various livestock species, significant discoveries are still forthcoming. Effective use of these tools in conjunction with sequencing data is essential for pinpointing promising candidate genes. Thus, post-GWAS analyses are critical as they provide insights into the gene networks that influence dairy cattle body linear type traits, as revealed by association studies.

### 3.5. GWAS Studies Screening Genetic Markers for Body Linear Type Traits 

Recent years have seen a proliferation of GWASs focusing on body conformation traits of Chinese Holstein cattle. In general, these studies on dairy cows have extensively explored various traits including body size, leg and feet conformation, rump, and mammary system traits. Across these studies, the sample sizes of animals typically range from 421 to 4841, likely due to the high labor costs required to measure body conformation traits (Table 5, Table 6, Table 7 and Table 8). Most studies employ high-density (HD) chips or sequencing technologies for genotyping and imputation, with HD panels being commonly used for imputation [26,97,98]. For body size traits, research on Holstein cattle in Canada and Chinese Holsteins commonly utilized the MLM and the GLM. For example, significant studies in Canada used sample sizes of up to 3577 Holstein cattle genotyped with 719,200 SNPs, achieving highly significant results [26]. In leg and feet conformation traits studies, the Gene Seek Genomic Profiler Bovine 100 K and Illumina 54 K were frequently used, with imputation relying on mutual or HD panels [58,99,100]. These studies predominantly employed the SMMA (Single Marker Mixed Model Analysis) and Farm CPU (Compressed Mixed Linear Model) models, both of which effectively manage large datasets and complex trait analyses. Studies on rump traits also leveraged HD chips, focusing on Chinese Holsteins, with GLM and Farm CPU being the favored analytical models (Table 5, Table 6, Table 7 and Table 8).

**Figure 2:**
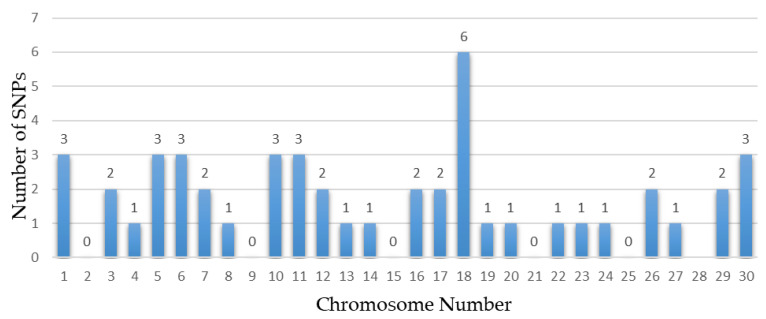
The number of significant SNPs associated with body size traits found in chromosomes from dairy cattle.

**Figure 3:**
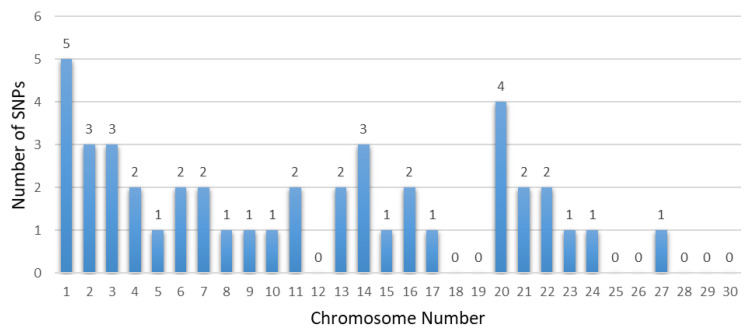
The number of significant SNPs associated with feet and leg conformation traits found in chromosomes from dairy cattle.

**Figure 4:**
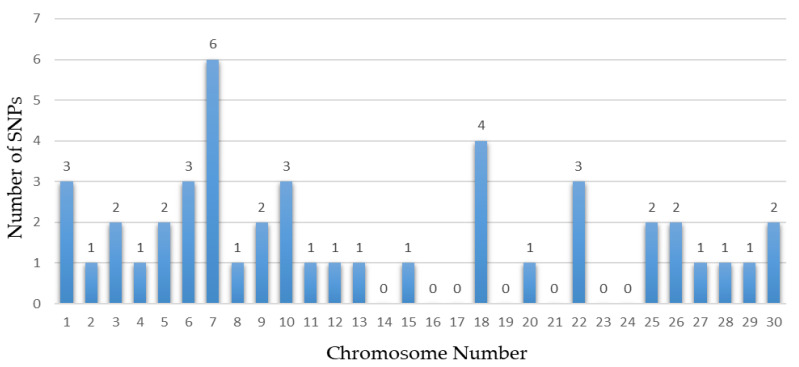
The number of significant SNPs associated with rump traits found in chromosomes from dairy cattle.

**Figure 5:**
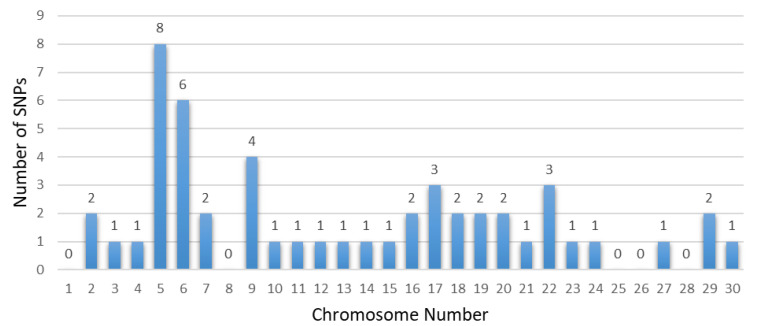
The number of significant SNPs associated with mammary system traits found in chromosomes from dairy cattle.

**Figure 6:**
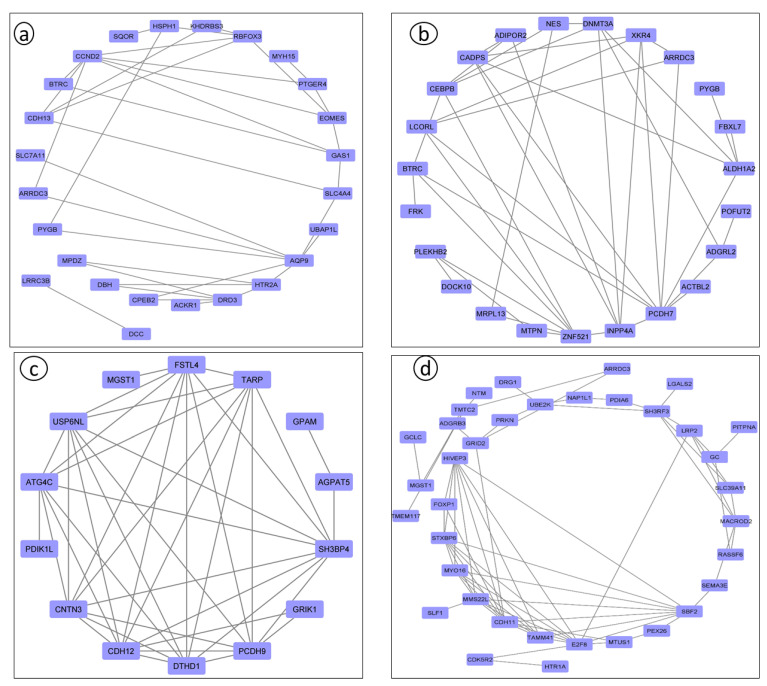
Protein–protein interactions between genes associated with body linear type traits. Note: (**a**) Body size traits; (**b**) feet and leg conformation traits; (**c**) rump traits; (**d**) mammary system traits.

Several chromosomes and genes repeatedly appeared across these studies, highlighting their significance in the genetic architecture of these traits. For instance, chromosome 5 frequently surfaced in body size traits, associated with the gene *CCND2*, showing significant markers like rs133960300 and rs109685956 with *p* values around 2.94 × 10^−9^ [26]. Chromosome 7 also appeared multiple times, particularly in leg and feet conformation traits, with the gene *ARRDC3* and *SLF1* showing markers such as rs109901274 and rs109618368, each with *p* values of 1.47 × 10^−9^ [26,60]. Additionally, genes like *KCNS3* on chromosome 11 and *ARRDC3* on chromosome 7 were recurrently identified in different studies, underscoring their crucial role in determining these traits [26,101]. Among them, some research reports are worthy of our attention. Yan et al. involved 445 Chinese Holstein cows, genotyped using a GGP BovineLD V3 SNP chip containing 26,151 public SNPs [101]. Additionally, Wang et al. conducted a GWAS using the Illumina Bovine HD 100 K BeadChip, identifying multiple candidate SNPs and genes associated with the body form composite index and feet and leg conformation traits in Holstein cattle [97]. Recent years have seen a proliferation of studies based on GWASs focusing on body conformation traits of Chinese Holstein cattle. Abdalla et al. reported 20 significant SNPs and 20 candidate genes associated with feet and leg conformation traits. In subsequent research, they reported 11 significant SNPs and 12 promising candidate genes, and emphasized the importance of rump traits, identifying 11 significant SNPs linked to reproductive and body shape-related traits [59]. Nazar et al. found 11 SNPs and 11 candidate genes related to mammary system teat shape conformation traits, including *MMS22L*, *E2F8*, *CSRP3*, and others. Furthermore, they identified numerous SNPs and candidate genes potentially linked to udder conformation traits, such as *MGST1*, *MGST2*, and *MTUS1* [60]. These findings collectively provide a comprehensive understanding of the genetic underpinnings of important traits in dairy cows, emphasizing the extensive datasets and advanced GWAS models employed. The results from these studies offer valuable insights for enhancing genetic selection and breeding strategies, ultimately contributing to the optimization of dairy cow productivity and health. Table 5, Table 6, Table 7 and Table 8 summarize the genetic markers associated with linear body traits in dairy cows, covering body size traits (Table 5), feet and leg conformation traits (Table 6), rump traits (Table 7), and mammary system traits (Table 8). Figure 2, Figure 3, Figure 4 and Figure 5 illustrate the number of significant SNPs associated with these traits, including body size traits (Figure 2), feet and leg conformation traits (Figure 3), rump traits (Figure 4), and mammary system traits (Figure 5). Additionally, Figure 6 summarizes the key genes associated with these traits.

### 3.6. Future Applications of the GWAS Strategy for Improving Body Conformation

Recent genomic studies have shown that predictions of livestock productivity can be redefined based on genomic and phenotypic data, challenging the traditional perspective on dairy cattle selection. The understanding of the natural variation in body linear type traits has significantly advanced in recent decades. With advancements in genomic sequencing, high-throughput SNP genotyping, and a wealth of genetic resources in databases such as The Bovine Genome Database (bovinegeNome.elsiklab.missouri.edu, accessed on 1 May 2024) and Cattle QTLdb (animalgeNome.org, accessed on 1 May 2024), a GWAS for body linear type traits is poised to become more informative.

GWAS findings can be applied in various ways, including in breeding programs, for the identification of candidate genes, genetic mapping, and gene editing. Furthermore, highly precise phenotyping by scientists and through high-throughput platforms will enhance a GWAS’s ability to identify novel loci. In the dairy industry, these advancements provide valuable resources that support and streamline breeding, genomics, and the genetic analysis of economically important traits.

A deeper analysis of the causal loci detected by a GWAS, such as haplotype-based analysis, is crucial for genomics-assisted dairy cattle breeding. A GWAS offers higher resolution due to more frequent recombination events and allows for the use of a broader genetic base compared to QTL mapping. For future research on body linear type traits, a GWAS should be considered an exploratory tool for selecting optimal parents for genomic selection and for further genetic and molecular validation of associations.

The analysis of complex traits in dairy cattle is expected to improve significantly with the help of statisticians and bioinformaticians. To facilitate this, further development of databases and statistical models is necessary. The integration of omics with genetics is critical for the improvement and molecular analysis of dairy cattle. Extending the study of natural variation to molecular mechanisms will provide deeper insights into the processes involved in cattle breeding.

## 4. Conclusions

In this review, we documented several genes associated with body linear type traits in dairy cattle. Furthermore, numerous SNPs within these candidate genes were highlighted, providing novel insights into the molecular basis of breeding and valuable information for understanding the genetic architecture of these traits in dairy cattle. Major SNPs identified on chromosomes 18, 1, 7, and 5 have been linked to body size, feet and leg conformation, rump, and mammary system traits, respectively. However, analyses of gene interactions have revealed connections between genes from different studies across these four groups of body linear type traits. Future research is essential to identify the specific genes and mutations involved. Additional studies are also needed to explore the biological functions and molecular regulatory networks of these genes.

## Figures and Tables

**Table 1:** Estimated values of heritability for body size traits.

Trait	Heritability	Breed	Number	Country	Ref.
ANG	0.10 ± 0.02	Holstein-Friesian	10,860	Serbia	[53]
ANG	0.22 ± 0.02	Serbian Holstein	32,512	Serbia	[54]
ANG	0.08 ± 0.01	Italian Jersey	6853	Italy	[51]
ANG	0.10 ± 0.08	Italian Holstein	253,602	Italy	[55]
ANG	0.48 ± 0.03	Chinese Holstein	1000	China	[56]
ANG	0.26 ± 0.02	Holstein	4841	Canada	[26]
BH	0.32 ± 0.03	Serbian Holstein	32,512	Serbia	[54]
BH	0.32 ± 0.03	Italian Jersey	6853	Italy	[51]
BH	0.30 ± 0.04	Chinese Holstein	7923	China	[49]
BH	0.56 ± 0.12	Dual-Purpose Simmental	1000	China	[57]
BH	0.33 ± 0.02	Chinese Holstein	45,517	China	[52]
BH	0.53 ± 0.12	Holstein	4841	Canada	[26]
CW	0.15 ± 0.02	Serbian Holstein	32,512	Serbia	[54]
CW	0.12 ± 0.02	Italian Jersey	6853	Italy	[51]
CW	0.24 ± 0.04	Chinese Holstein	7923	China	[49]
CW	0.13 ± 0.08	Dual-Purpose Simmental	1000	China	[57]
CW	0.08 ± 0.01	Chinese Holstein	45,517	China	[52]
CW	0.17 ± 0.01	Chinese Holstein	1000	China	[56]
CW	0.22 ± 0.02	Holstein	4841	Canada	[26]
BD	0.17 ± 0.02	Serbian Holstein	32,512	Serbia	[54]
BD	0.12 ± 0.02	Italian Jersey	6853	Italy	[51]
BD	0.12 ± 0.02	Chinese Holstein	7923	China	[49]
BD	0.17 ± 0.08	Dual-Purpose Simmental	1000	China	[57]
BD	0.14 ± 0.01	Chinese Holstein	45,517	China	[52]
BD	0.10 ± 0.01	Chinese Holstein	1000	China	[56]
BD	0.32 ± 0.02	Holstein	4841	Canada	[26]

Abbreviations: body height, BH; body depth, BD; chest width, CW; angularity, ANG.

**Table 2:** Estimated values of heritability for feet and leg conformation traits.

Trait	Heritability	Breed	Number	Country	Ref.
HD	0.15 ± 0.01	Chinese Holstein	1000	China	[58]
HD	0.37 ± 0.05	Chinese Holstein	7923	China	[49]
HD	0.05 ± 0.05	Dual-Purpose Simmental	1000	China	[57]
HD	0.02 ± 0.01	Chinese Holstein	45,517	China	[52]
HD	0.08 ± 0.00	Holstein	4841	Canada	[26]
BQ	0.05 ± 0.00	Chinese Holstein	1000	China	[58]
BQ	0.37 ± 0.04	Chinese Holstein	7923	China	[49]
BQ	0.07 ± 0.05	Dual-Purpose Simmental	1000	China	[57]
BQ	0.05 ± 0.01	Chinese Holstein	45,517	China	[52]
BQ	0.30 ± 0.03	Holstein	4841	Canada	[26]
RLSV	0.17 ± 0.01	Chinese Holstein	1000	China	[58]
RLSV	0.10 ± 0.02	Serbian Holstein	32,512	Serbia	[54]
RLSV	0.04 ± 0.00	Italian Jersey	6853	Italy	[51]
RLSV	0.09 ± 0.06	Dual-Purpose Simmental	1000	China	[57]
RLSV	0.04 ± 0.01	Chinese Holstein	45,517	China	[52]
RLSV	0.24 ± 0.03	Holstein	4841	Canada	[26]
RLRV	0.15 ± 0.01	Chinese Holstein	1000	China	[58]
RLRV	0.16 ± 0.02	Serbian Holstein	10,860	Serbia	[53]
RLRV	0.13 ± 0.01	Holstein	4841	Canada	[26]
RLRV	0.04 ± 0.00	Italian Jersey	6853	Italy	[51]
RLRV	0.37 ± 0.04	Chinese Holstein	7923	China	[49]
RLRV	0.12 ± 0.07	Dual-Purpose Simmental	1000	China	[57]
RLRV	0.06 ± 0.01	Chinese Holstein	45,517	China	[52]
FA	0.14 ± 0.02	Serbian Holstein	32,512	Serbia	[54]
FA	0.07 ± 0.01	Italian Jersey	6853	Italy	[51]
FA	0.14 ± 0.03	Chinese Holstein	7923	China	[49]
FA	0.11 ± 0.06	Dual-Purpose Simmental	1000	China	[57]
FA	0.04 ± 0.01	Chinese Holstein	45,517	China	[52]
FA	0.11 ± 0.01	Holstein	4841	Canada	[26]

Abbreviations: heel depth, HD; bone quality, BQ; rear leg side view, RLSV; rear leg rear view, RLRV; foot angle, FA.

**Table 3:** Estimated values of heritability for rump traits.

Trait	Heritability	Breed	Number	Country	Ref.
RP	0.16 ± 0.02	Serbian Holstein	10,860	Serbia	[53]
RW	0.18 ± 0.02	Serbian Holstein	32,512	Serbia	[54]
RW	0.06 ± 0.00	Italian Jersey	6853	Italy	[51]
RW	0.28 ± 0.04	Chinese Holstein	7923	China	[49]
RW	0.22 ± 0.09	Dual-Purpose Simmental	1000	China	[57]
RW	0.08 ± 0.01	Chinese Holstein	45,517	China	[52]
RW	0.20 ± 0.02	Chinese Holstein	1000	China	[59]
RW	0.34 ± 0.03	Holstein	4841	Canada	[26]
RA	0.14 ± 0.02	Italian Jersey	6853	Italy	[51]
RA	0.15 ± 0.07	Dual-Purpose Simmental	1000	China	[57]
RA	0.11 ± 0.01	Chinese Holstein	45,517	China	[52]
RA	0.22 ± 0.02	Chinese Holstein	1000	China	[59]
RA	0.37 ± 0.03	Holstein	4841	Canada	[26]
LS	0.32 ± 0.04	Chinese Holstein	1000	China	[49]
LS	0.38 ± 0.05	Chinese Holstein	1000	China	[59]
LS	0.25 ± 0.02	Holstein	4841	Canada	[26]
PS	0.18 ± 0.03	Chinese Holstein	7923	China	[49]
PS	0.09 ± 0.01	Holstein	4841	Canada	[26]
RL	0.29 ± 0.11	Dual-Purpose Simmental	1000	China	[57]

Abbreviations: rump position, RP; rump angle, RA; lion strength, LS; pin setting, PS; rump width, RW; rump length, RL.

**Table 4:** Estimated values of heritability for mammary system traits.

Trait	Heritability	Breed	Number	Country	Ref.
FUA	0.11 ± 0.02	Holstein-Friesian	10,860	Serbia	[53]
FUA	0.18 ± 0.02	Serbian Holstein	32,512	Serbia	[54]
FUA	0.16 ± 0.02	Italian Jersey	6853	Italy	[51]
FUA	0.19 ± 0.08	Dual-Purpose Simmental	1000	China	[57]
FUA	0.11 ± 0.01	Chinese Holstein	45,517	China	[52]
FUA	0.04 ± 0.00	Chinese Holstein	1000	China	[60]
FUA	0.28 ± 0.02	Holstein	4841	Canada	[26]
AUA	0.24 ± 0.02	Chinese Holstein	1000	China	[60]
FTP	0.07 ± 0.01	Holstein-Friesian	10,860	Serbia	[53]
FTP	0.13 ± 0.02	Serbian Holstein	32,512	Serbia	[54]
FTP	0.08 ± 0.01	Italian Jersey	6853	Italy	[51]
FTP	0.14 ± 0.03	Chinese Holstein	7923	China	[49]
FTP	0.20 ± 0.08	Dual-Purpose Simmental	1000	China	[57]
FTP	0.37 ± 0.04	Chinese Holstein	1000	China	[58]
FTP	0.31 ± 0.03	Holstein	4841	Canada	[26]
FTP	0.07 ± 0.01	Chinese Holstein	45,517	China	[52]
FTL	0.16 ± 0.02	Serbian Holstein	32,512	Serbia	[54]
FTL	0.10 ± 0.02	Italian Jersey	6853	Italy	[51]
FTL	0.28 ± 0.04	Chinese Holstein	7923	China	[49]
FTL	0.12 ± 0.06	Dual-Purpose Simmental	1000	China	[57]
FTL	0.05 ± 0.01	Chinese Holstein	45,517	China	[52]
TL	0.06 ± 0.01	Holstein-Friesian	10,860	Serbia	[1]
TL	0.29 ± 0.02	Holstein	4841	Canada	[26]
FTL	0.13 ± 0.01	Chinese Holstein	1000	China	[58]
UD	0.08 ± 0.01	Holstein-Friesian	10,860	Serbia	[53]
UD	0.22 ± 0.02	Italian Jersey	6853	Italy	[51]
UD	0.21 ± 0.03	Chinese Holstein	7923	China	[49]
UD	0.22 ± 0.09	Dual-Purpose Simmental	1000	China	[57]
UD	0.12 ± 0.01	Chinese Holstein	45,517	China	[52]
UD	0.49 ± 0.03	Chinese Holstein	1000	China	[60]
UD	0.46 ± 0.03	Holstein	4841	Canada	[26]
RUH	0.08 ± 0.01	Holstein-Friesian	10,860	Serbia	[53]
RUH	0.17 ± 0.07	Dual-Purpose Simmental	1000	China	[57]
RUH	0.10 ± 0.01	Chinese Holstein	45,517	China	[52]
RUH	0.23 ± 0.02	Holstein	4841	Canada	[26]
MSL	0.10 ± 0.04	Chinese Holstein	7923	China	[49]
CSL	0.34 ± 0.03	Chinese Holstein	1000	China	[60]
CSL	0.14 ± 0.01	Holstein	4841	Canada	[26]

Abbreviations: central suspensory ligament, CSL; teat length, TL; fore teat length, FTL; anterior udder attachment, AUA; udder depth, UD; fore udder attachment, FUA; fore teats placement, FTP; rear udder height, RUH; median suspensory, MSL.

**Table 5:** GWAS study for screening genetic markers associated with body size traits.

Traits	SNPs	Genes	Chr.	*p* Value	Genotype	Imputed	SNP Size	Sample Size	Breed	Model	Country	Ref.
BH	rs133960300	*CCND2*	5	2.94 × 10^−9^	Illumina 50 K/HD	HD panel	719,200	3577	Holstein	MLM	Canada	[26]
	rs109685956	*CCND2*	5	2.94 × 10^−9^	Illumina 50 K/HD	HD panel	719,200	3577	Holstein	MLM	Canada	[26]
	rs109882115	*ENSBTAG00000039491*	18	1.19 × 10^−9^	Illumina 50 K/HD	HD panel	719,200	3577	Holstein	MLM	Canada	[26]
	rs109478645	*ENSBTAG00000037537*	18	1.22 × 10^−9^	Illumina 50 K/HD	HD panel	719,200	3577	Holstein	MLM	Canada	[26]
	ARS-BFGL-NGS-41612	*KCNS3*	11	4.93 × 10^−7^	Bovine LD V3 SNP	No	20,632	421	Chinese Holstein	MLM	China	[101]
	BovineHD1100030541	*LOC789076*	11	1.5 × 10^−6^	Bovine LD V3 SNP	No	20,632	421	Chinese Holstein	MLM	China	[101]
	BovineHD2300011340	*NHLRC1*	23	2.39 × 10^−6^	Bovine LD V3 SNP	No	20,632	421	Chinese Holstein	MLM	China	[101]
	Hapmap38550-BTA-98603	*LRRC3B*	27	1.66 × 10^−6^	Bovine LD V3 SNP	No	20,632	421	Chinese Holstein	MLM	China	[101]
	Hapmap60794-rs29022851	*CPEB2*	6	9.53 × 10^−3^	BovineSNP50 Bead Chip/Illumina 54 K	Mutual	52,166	1314	Chinese Holstein	SMMA	China	[99]
	BTA-72885-no-rs	*LOC782090*	29	9.69 × 10^−3^	BovineSNP50 Bead Chip/Illumina 54 K	Mutual	52,166	1314	Chinese Holstein	SMMA	China	[99]
	rs110462304	*MYH15*	1	1.86 × 10^−7^	Gene Seek Genomic Profiler Bovine 100 K	No	84,406	984	Chinese Holstein	Farm CPU	China	[56]
	rs109930583	*C6H4orf17*	6	2.06 × 10^−7^	Gene Seek Genomic Profiler Bovine 100 K	No	84,406	984	Chinese Holstein	Farm CPU	China	[56]
	rs109824125	*KHDRBS3*	14	4.98 × 10^−7^	Gene Seek Genomic Profiler Bovine 100 K	No	84,406	984	Chinese Holstein	Farm CPU	China	[56]
	rs42188649	*AIP*	29	5.80 × 10^−7^	Gene Seek Genomic Profiler Bovine 100 K	No	84,406	984	Chinese Holstein	Farm CPU	China	[56]
	Hapmap60480-ss46526970	*NDUFA9, KCNA1*	5	1.18 × 10^−5^	BovineSNP50 Bead Chip/Illumina 54 K	Mutual	38,720	2329	Korean Holstein	MLM	Korea	[100]
BD	rs109478645	*ENSBTAG00000037537*	18	2.29 × 10^−22^	BovineSNP50 Bead Chip/Illumina 50 K	HD panel	601,717	4841	Holstein	MLM	Canada	[26]
	rs110801791	*CTU1*	18	9.73 × 10^−20^	BovineSNP50 Bead Chip/Illumina 50 K	HD panel	601,717	4841	Holstein	MLM	Canada	[26]
	rs135253383	*CTU1*	18	1.03 × 10^−19^	BovineSNP50 Bead Chip/Illumina 50 K	HD panel	601,717	4841	Holstein	MLM	Canada	[26]
	Hapmap40339-BTA-117016	*DARC*	3	8.72 × 10^−3^	BovineSNP50 Bead Chip/Illumina 54 K	Mutual	52,166	1314	Chinese Holstein	SMMA	China	[99]
	rs133735152	*DCC*	24	2.33 × 10^−8^	Gene Seek Genomic Profiler Bovine 100 K	No	84,406	984	Chinese Holstein	Farm CPU	China	[56]
	rs43286429	*LOC112447004*	1	4.71 × 10^−7^	Gene Seek Genomic Profiler Bovine 100 K	No	84,406	984	Chinese Holstein	Farm CPU	China	[56]
	BTB-00853109	*CCDC12, PTH1R*	22	1.99 × 10^−5^	BovineSNP50 Bead Chip/Illumina 54 K	Mutual	38,720	2329	Korean Holstein	MLM	Korea	[100]
	Hapmap43881-BTA-54837	*PRSS45, PRSS46*	22	2.02 × 10^−5^	BovineSNP50 Bead Chip/Illumina 54 K	Mutual	38,720	2329	Korean Holstein	MLM	Korea	[100]
CW	rs109901274	*ARRDC3*	7	1.47 × 10^−9^	BovineSNP50 Bead Chip/Illumina 50 K	HD panel	601,717	4841	Holstein	MLM	Canada	[26]
	rs109618368	*ARRDC3*	7	1.47 × 10^−9^	BovineSNP50 Bead Chip/Illumina 50 K	HD panel	601,717	4841	Holstein	MLM	Canada	[26]
	BovineHD1700010514	*LOC512119*	17	1.34 × 10^−7^	Bovine LD V3 SNP	No	20,632	421	Chinese Holstein	MLM	China	[101]
	BTA-110160-no-rs	*GAS1*	8	9.69 × 10^−3^	BovineSNP50 Bead Chip/Illumina 54 K	Mutual	52,166	1314	Chinese Holstein	SMMA	China	[99]
	ARS-BFGL-NGS-115466	*CDH13*	18	9.69 × 10^−3^	BovineSNP50 Bead Chip/Illumina 54 K	Mutual	52,166	1314	Chinese Holstein	SMMA	China	[99]
	BTA-45515-no-rs	*PTRF*	19	9.69 × 10^−3^	BovineSNP50 Bead Chip/Illumina 54 K	Mutual	52,166	1314	Chinese Holstein	SMMA	China	[99]
	BTB-00922140	*POU6F2*	4	9.69 × 10^−3^	BovineSNP50 Bead Chip/Illumina 54 K	Mutual	52,166	1314	Chinese Holstein	SMMA	China	[99]
	rs110355602	*SQOR*	10	9.45 × 10^−11^	Gene Seek Genomic Profiler Bovine 100 K	No	84,406	984	Chinese Holstein	Farm CPU	China	[56]
	rs43615333	*UBAP1L*	10	1.17 × 10^−7^	Gene Seek Genomic Profiler Bovine 100 K	No	84,406	984	Chinese Holstein	Farm CPU	China	[56]
	rs42095998	*VTI1A*	26	8.22 × 10^−7^	Gene Seek Genomic Profiler Bovine 100 K	No	84,406	984	Chinese Holstein	Farm CPU	China	[56]
	BTB-00853109	*CCDC12, PTH1R*	22	1.52 × 10^−5^	BovineSNP50 Bead Chip/Illumina 54 K	Mutual	38,720	2329	Korean Holstein	MLM	Korea	[100]
ANG	rs109512265	*SLC4A4*	6	1.51 × 10^−8^	BovineSNP50 Bead Chip/Illumina 50 K	HD panel	601,717	4841	Holstein	MLM	Canada	[26]
	BTA-116883-no-rs	*LOC786124*	30	1.56 × 10^−6^	Bovine LD V3 SNP	No	20,632	421	Chinese Holstein	MLM	China	[101]
	BovineHD3000032546	*LOC537655*	30	6.55 × 10^−7^	Bovine LD V3 SNP	No	20,632	421	Chinese Holstein	MLM	China	[101]
	BovineHD3000037672	*LOC786725*	30	5.10 × 10^−8^	Bovine LD V3 SNP	No	20,632	421	Chinese Holstein	MLM	China	[101]
	ARS-BFGL-NGS-14022	*SLC25A24*	3	9.69 × 10^−3^	BovineSNP50 Bead Chip/Illumina 54 K	Mutual	52,166	1314	Chinese Holstein	SMMA	China	[99]
	ARS-BFGL-NGS-113826	*HTR2A*	12	9.69 × 10^−3^	BovineSNP50 Bead Chip/Illumina 54 K	Mutual	52,166	1314	Chinese Holstein	SMMA	China	[99]
	rs135918869	*CCDC59*	5	1.32 × 10^−7^	Gene Seek Genomic Profiler Bovine 100 K.	No	84,406	984	Chinese Holstein	Farm CPU	China	[56]
	BTA-67308-no-rs	*GNAI3*	3	6.32 × 10^−6^	BovineSNP50 Bead Chip/Illumina 54 K	Mutual	38,720	2329	Korean Holstein	MLM	Korea	[100]
	ARS-BFGL-NGS-5218	*AP3B1*	10	2.31 × 10^−5^	BovineSNP50 Bead Chip/Illumina 54 K	Mutual	38,720	2329	Korean Holstein	MLM	Korea	[100]
Body size	rs137415420	*DRD3*	1	5.57 × 10^−10^	Illumina 50 K/HD	Mutual	598,016	4578	Brown Swiss	GLM	Switzerland	[98]
	rs110574932	*DBH*	11	5.63 × 10^−8^	Illumina 50 K/HD	Mutual	598,016	4578	Brown Swiss	GLM	Switzerland	[98]
	rs42088986	*BTRC*	26	1.00 × 10^−14^	Illumina 50 K/HD	Mutual	598,016	4578	Brown Swiss	GLM	Switzerland	[98]
BFCI	ARS–BFGL–NGS−39319	*MPDZ*	8	4.59 × 10^−8^	Illumina Bovine HD100 k Bead Chip	No	95,256	1313	Holstein	Farm CPU	China	[97]
	BovineHD1000015574	*AQP9*	10	3.07 × 10^−9^	Illumina Bovine HD100 k Bead Chip	No	95,256	1313	Holstein	Farm CPU	China	[97]
	BovineHD1200008803	*HSPH1*	12	3.72 × 10^−8^	Illumina Bovine HD100 k Bead Chip	No	95,256	1313	Holstein	Farm CPU	China	[97]
	BovineHD1300012605	*PYGB*	13	8.06 × 10^−8^	Illumina Bovine HD100 k Bead Chip	No	95,256	1313	Holstein	Farm CPU	China	[97]
	ARS–BFGL–NGS−66252	*MMEL1*	16	2.79 × 10^−8^	Illumina Bovine HD100 k Bead Chip	No	95,256	1313	Holstein	Farm CPU	China	[97]
	BovineHD1600023101	*ATP6V1G3*	16	9.44 × 10^−8^	Illumina Bovine HD100 k Bead Chip	No	95,256	1313	Holstein	Farm CPU	China	[97]
	BovineHD1700005623	*SLC7A11*	17	2.82 × 10^−8^	Illumina Bovine HD100 k Bead Chip	No	95,256	1313	Holstein	Farm CPU	China	[97]
	BovineHD1900015024	*RBFOX3*	19	4.34 × 10^−7^	Illumina Bovine HD100 k Bead Chip	No	95,256	1313	Holstein	Farm CPU	China	[97]
	BTA−50244–no–rs	*PTGER4*	20	5.84 × 10^−13^	Illumina Bovine HD100 k Bead Chip	No	95,256	1313	Holstein	Farm CPU	China	[97]
	BovineHD2200000513	*EOMES*	22	2.09 × 10^−7^	Illumina Bovine HD100 k Bead Chip	No	95,256	1313	Holstein	Farm CPU	China	[97]

Abbreviations: body height, BH; body depth, BD; chest width, CW; angularity, ANG; mixed linear model, MLM; generalized linear model, GLM; single marker mixed model analysis, SMMA; reference, Ref; body form composite index, BFCI.

**Table 6:** GWAS study for screening genetic markers associated with feet and leg conformation traits.

Traits	SNPs	Genes	Chr.	*p* Value	Genotype	Imputed	SNP Size	Sample Size	Breed	Model	Country	Ref.
BQ	rs109901274	*ARRDC3*	7	9.10 × 10^−11^	Bovine HD	HD panel	601,717	4841	Holstein	MLM	Canada	[26]
	rs109618368	*ARRDC3*	7	9.10 × 10^−11^	Bovine HD	HD panel	601,717	4841	Holstein	MLM	Canada	[26]
	BTA-87372-no-rs	*LOC100337296*	1	9.49 × 10^−3^	Gene Seek Genomic Profiler Bovine 100 K	No	84,906	984	Chinese Holstein	Farm CPU	China	[58]
	BTA-117758-no-rs	*C8H9orf30*	15	9.49 × 10^−3^	Gene Seek Genomic Profiler Bovine 100 K	No	84,906	984	Chinese Holstein	Farm CPU	China	[58]
	rs29015846	*LOC112447952*	8	1.99 × 10^−7^	Gene Seek Genomic Profiler Bovine 100 K	No	84,906	984	Chinese Holstein	Farm CPU	China	[58]
	rs133088614	*TMEM229A*	4	2.25 × 10^−7^	Gene Seek Genomic Profiler Bovine 100 K	No	84,906	984	Chinese Holstein	Farm CPU	China	[58]
	rs41845981	*POLE*	17	3.14 × 10^−7^	Gene Seek Genomic Profiler Bovine 100 K	No	84,906	984	Chinese Holstein	Farm CPU	China	[58]
	rs136017102	*XKR4*	14	6.22 × 10^−7^	Gene Seek Genomic Profiler Bovine 100 K	No	84,906	984	Chinese Holstein	Farm CPU	China	[58]
	rs110949452	*CADPS*	22	7.67 × 10^−7^	Gene Seek Genomic Profiler Bovine 100 K	No	84,906	984	Chinese Holstein	Farm CPU	China	[58]
	ARS-BFGL-NGS-37048	*EVX1, HOXA13*	4	1.49 × 10^−5^	BovineSNP50 Bead Chip/Illumina 54 K	Mutual	38,720	2329	Korean Holstein	MLM	Korea	[100]
	Hapmap54735-ss46526095	*VAMP4*	16	4.31 × 10^−6^	BovineSNP50 Bead Chip/Illumina 54 K	Mutual	38,720	2329	Korean Holstein	MLM	Korea	[100]
FA	ARS-BFGL-NGS-18261	*PLEKHB2*	2	9.29 × 10^−3^	BovineSNP50 Bead Chip/Illumina 54 K	Mutual	52,166	1314	Chinese Holstein	SMMA	China	[99]
	ARS-BFGL-NGS-73625	*NES*	3	9.29 × 10^−3^	BovineSNP50 Bead Chip/Illumina 54 K	Mutual	52,166	1314	Chinese Holstein	SMMA	China	[99]
	Hapmap48448-BTA-71823	*MTPN*	4	9.29 × 10^−3^	BovineSNP50 Bead Chip/Illumina 54 K	Mutual	52,166	1314	Chinese Holstein	SMMA	China	[99]
RLSV	ARS-BFGL-NGS-97763	*DOCK10*	2	9.42 × 10^−3^	BovineSNP50 Bead Chip/Illumina 54 K	Mutual	52,166	1314	Chinese Holstein	SMMA	China	[99]
	Hapmap29973-BTA-129162	*PAG1*	14	9.42 × 10^−3^	BovineSNP50 Bead Chip/Illumina 54 K	Mutual	52,166	1314	Chinese Holstein	SMMA	China	[99]
	UA-IFASA-4800	*ZNF521*	24	9.42 × 10^−3^	BovineSNP50 Bead Chip/Illumina 54 K	Mutual	52,166	1314	Chinese Holstein	SMMA	China	[99]
	rs41565304	*ADIPOR2*	5	1.11 × 10^−9^	Gene Seek Genomic Profiler Bovine 100 K	No	84,906	984	Chinese Holstein	Farm CPU	China	[58]
	rs43656945	*INPP4A*	11	2.32 × 10^−7^	Gene Seek Genomic Profiler Bovine 100 K	No	84,906	984	Chinese Holstein	Farm CPU	China	[58]
	rs136593856	*DNMT3A*	11	5.07 × 10^−7^	Gene Seek Genomic Profiler Bovine 100 K	No	84,906	984	Chinese Holstein	Farm CPU	China	[58]
	rs42791722	*ALDH1A2*	10	7.40 × 10^−7^	Gene Seek Genomic Profiler Bovine 100 K	No	84,906	984	Chinese Holstein	Farm CPU	China	[58]
	rs42639670	*PCDH7*	6	9.65 × 10^−7^	Gene Seek Genomic Profiler Bovine 100 K	No	84,906	984	Chinese Holstein	Farm CPU	China	[58]
RLRV	rs134130409	*BARHL2*	3	6.72 × 10^−8^	Gene Seek Genomic Profiler Bovine 100 K	No	84,906	984	Chinese Holstein	Farm CPU	China	[58]
	rs134139959	*FBXL7*	20	1.11 × 10^−7^	Gene Seek Genomic Profiler Bovine 100 K	No	84,906	984	Chinese Holstein	Farm CPU	China	[58]
	rs41638134	*LOC107132214*	1	6.11 × 10^−7^	Gene Seek Genomic Profiler Bovine 100 K	No	84,906	984	Chinese Holstein	Farm CPU	China	[58]
	ARS-BFGL-NGS-629	*MALRD1*	13	1.05 × 10^−5^	BovineSNP50 Bead Chip/Illumina 54 K	Mutual	38,720	2329	Korean Holstein	MLM	Korea	[100]
HD	rs137022628	*ACTBL2*	20	3.03 × 10^−9^	Gene Seek Genomic Profiler Bovine 100 K	No	84,906	984	Chinese Holstein	Farm CPU	China	[58]
	rs109652453	*SYCP2L*	23	4.22 × 10^−8^	Gene Seek Genomic Profiler Bovine 100 K	No	84,906	984	Chinese Holstein	Farm CPU	China	[58]
	rs42110372	*LOC112444670*	27	3.11 × 10^−7^	Gene Seek Genomic Profiler Bovine 100 K	No	84,906	984	Chinese Holstein	Farm CPU	China	[58]
	rs109601642	*LOC101907219*	20	4.64 × 10^−7^	Gene Seek Genomic Profiler Bovine 100 K	No	84,906	984	Chinese Holstein	Farm CPU	China	[58]
	rs41577664	*LOC112441589*	15	7.43 × 10^−7^	Gene Seek Genomic Profiler Bovine 100 K	No	84,906	984	Chinese Holstein	Farm CPU	China	[58]
	rs134726669	*MRPL13*	14	7.59 × 10^−7^	Gene Seek Genomic Profiler Bovine 100 K	No	84,906	984	Chinese Holstein	Farm CPU	China	[58]
	BTB-01928726	*INHBA*	4	2.08 × 10^−5^	BovineSNP50 Bead Chip/Illumina 54 K	Mutual	38,720	2329	Korean Holstein	MLM	Korea	[100]
LC	rs110597649	*RSPO4*	2	4.13 × 10^−13^	Illumina 50 K/HD	HD panel	598,016	4578	Brown Swiss	GLM	Switzerland	[98]
	rs134127590	*BTRC*	1	5.92 × 10^−10^	Illumina 50 K/HD	HD panel	598,016	4578	Brown Swiss	GLM	Switzerland	[98]
FTLEG	BovineHD0100020157	*SNX4*	1	2.07 × 10^−7^	Illumina Bovine HD100 k Bead Chip	No	95,256	1313	Holstein	Farm CPU	China	[97]
	ARS–BFGL–NGS−56584	*POFUT2*	1	7.56 × 10^−8^	Illumina Bovine HD100 k Bead Chip	No	95,256	1313	Holstein	Farm CPU	China	[97]
	BovineHD0300019080	*ADGRL2*	3	1.06 × 10^−8^	Illumina Bovine HD100 k Bead Chip	No	95,256	1313	Holstein	Farm CPU	China	[97]
	BTB−01326707	*LCORL*	6	3.16 × 10^−11^	Illumina Bovine HD100 k Bead Chip	No	95,256	1313	Holstein	Farm CPU	China	[97]
	BTB−00124923	*FRK*	9	3.42 × 10^−7^	Illumina Bovine HD100 k Bead Chip	No	95,256	1313	Holstein	Farm CPU	China	[97]
	BovineHD1300012605	*PYGB*	13	3.23 × 10^−9^	Illumina Bovine HD100 k Bead Chip	No	95,256	1313	Holstein	Farm CPU	China	[97]
	Hapmap50322–BTA−34017	*CEBPB*	13	8.11 × 10^−8^	Illumina Bovine HD100 k Bead Chip	No	95,256	1313	Holstein	Farm CPU	China	[97]
	BovineHD1600000840	*KLHDC8A*	16	3.74 × 10^−7^	Illumina Bovine HD100 k Bead Chip	No	95,256	1313	Holstein	Farm CPU	China	[97]
	BovineHD1600008381	*TMEM63A*	16	7.79 × 10^−9^	Illumina Bovine HD100 k Bead Chip	No	95,256	1313	Holstein	Farm CPU	China	[97]
	BovineHD2000011811	*SUB1*	20	4.11 × 10^−11^	Illumina Bovine HD100 k Bead Chip	No	95,256	1313	Holstein	Farm CPU	China	[97]
	BTA−14388–rs29023151	*IL5RA*	22	8.59 × 10^−10^	Illumina Bovine HD100 k Bead Chip	No	95,256	1313	Holstein	Farm CPU	China	[97]

Abbreviations: single nucleotide polymorphism, SNP; chromosome, Chr.; bone quality, BQ; foot angle, FA; rear leg rear view, RLRV; rear leg side view, RLSV; heel depth, HD; leg conformation, LC; feet and leg conformation traits, FTLEG; mixed linear model, MLM; generalized linear model, GLM; single trait mixed model analysis, SMMA; reference, Ref.

**Table 7:** GWAS study for screening genetic markers associated with body rump traits.

Traits	SNPs	Genes	Chr.	*p* Value	Genotype	Imputed	SNP Size	Sample Size	Breed	Model	Country	Ref.
LS	BovineHD0500017277	*NEDD1*	5	2.90 × 10^−7^	50 K/HD	Mutual	598,016	4578	Chinese Holstein	GLM	China	[98]
	ARS-BFGL-NGS-20197	*HB6*	7	5.71 × 10^−7^	50 K/HD	Mutual	598,016	4578	Chinese Holstein	GLM	China	[98]
	BovineHD2800013502	*LOC100141022*	28	4.71 × 10^−7^	50 K/HD	Mutual	598,016	4578	Chinese Holstein	GLM	China	[98]
	ARS-BFGL-NGS-70552	*SERGEF*	15	8.95 × 10^−3^	BovineSNP50 Bead Chip/Illumina 54 K	Mutual	52,166	1314	Chinese Holstein	SMMA	China	[99]
	BTB-00938945	*GPAM*	26	8.95 × 10^−3^	BovineSNP50 Bead Chip/Illumina 54 K	Mutual	52,166	1314	Chinese Holstein	SMMA	China	[99]
	rs42946768	*CDH12*	20	3.08 × 10^−8^	GGP Bovine 100 K	No	84,407	984	Chinese Holstein	Farm CPU	China	[59]
	rs109073659	*PCDH9*	12	2.23 × 10^−7^	GGP Bovine 100 K	No	84,407	984	Chinese Holstein	Farm CPU	China	[59]
	rs43162548	*TARP*	4	2.99 × 10^−7^	GGP Bovine 100 K	No	84,407	984	Chinese Holstein	Farm CPU	China	[59]
	rs133475777	*DTHD1*	6	4.29 × 10^−7^	GGP Bovine 100 K	No	84,407	984	Chinese Holstein	Farm CPU	China	[59]
RA	BovineHD0100019488	*CCDC14*	1	4.88 × 10^−7^	Bovine LD V3 SNP	No	20,632	421	Chinese Holstein	MLM	China	[101]
	BTB-00003652	*GRIK1*	1	1.76 × 10^−6^	Bovine LD V3 SNP	No	20,632	421	Chinese Holstein	MLM	China	[101]
	BovineHD0100041062	*BACE2*	1	2.03 × 10^−6^	Bovine LD V3 SNP	No	20,632	421	Chinese Holstein	MLM	China	[101]
	BovineHD0200037025	*PDIK1L*	2	6.11 × 10^−7^	Bovine LD V3 SNP	No	20,632	421	Chinese Holstein	MLM	China	[101]
	Hapmap38371-BTA-105598	*AMBN*	6	1.58 × 10^−6^	Bovine LD V3 SNP	No	20,632	421	Chinese Holstein	MLM	China	[101]
	BovineHD0700024393	*MSH3*	7	4.32 × 10^−9^	Bovine LD V3 SNP	No	20,632	421	Chinese Holstein	MLM	China	[101]
	BovineHD0700024587	*SSBP2*	7	1.04 × 10^−7^	Bovine LD V3 SNP	No	20,632	421	Chinese Holstein	MLM	China	[101]
	BovineHD0800030195	*SVEP1*	8	2.25 × 10^−6^	Bovine LD V3 SNP	No	20,632	421	Chinese Holstein	MLM	China	[101]
	BTA-106078-no-rs	*HIVEP2*	9	9.84 × 10^−7^	Bovine LD V3 SNP	No	20,632	421	Chinese Holstein	MLM	China	[101]
	BovineHD1000013067	*MAP4K5*	10	8.09 × 10^−8^	Bovine LD V3 SNP	No	20,632	421	Chinese Holstein	MLM	China	[101]
	BovineHD1000018043	*SLC24A5*	10	7.73 × 10^−7^	Bovine LD V3 SNP	No	20,632	421	Chinese Holstein	MLM	China	[101]
	Hapmap49737-BTA-75278	*PRKCH*	10	6.00 × 10^−7^	Bovine LD V3 SNP	No	20,632	421	Chinese Holstein	MLM	China	[101]
	ARS-BFGL-NGS-116541	*LIG1*	18	2.37 × 10^−6^	Bovine LD V3 SNP	No	20,632	421	Chinese Holstein	MLM	China	[101]
	BovineHD1800016250	*SYNGR4*	18	7.28 × 10^−8^	Bovine LD V3 SNP	No	20,632	421	Chinese Holstein	MLM	China	[101]
	ARS-BFGL-NGS-31529	*LMTK3*	18	2.12 × 10^−6^	Bovine LD V3 SNP	No	20,632	421	Chinese Holstein	MLM	China	[101]
	BovineHD2200013812	*CACNA1D*	22	1.72 × 10^−6^	Bovine LD V3 SNP	No	20,632	421	Chinese Holstein	MLM	China	[101]
	BovineHD2200013926	*RFT1*	22	5.34 × 10^−7^	Bovine LD V3 SNP	No	20,632	421	Chinese Holstein	MLM	China	[101]
	ARS-BFGL-NGS-101981	*ADAP1*	25	1.32 × 10^−6^	Bovine LD V3 SNP	No	20,632	421	Chinese Holstein	MLM	China	[101]
	BovineHD2600004135	*LOC522146*	26	1.32 × 10^−6^	Bovine LD V3 SNP	No	20,632	421	Chinese Holstein	MLM	China	[101]
	BovineHD3000000680	*KLHL13*	30	2.28 × 10^−6^	Bovine LD V3 SNP	No	20,632	421	Chinese Holstein	MLM	China	[101]
	BTA-21001-no-rs	*MSL3*	30	2.28 × 10^−6^	Bovine LD V3 SNP	No	20,632	421	Chinese Holstein	MLM	China	[101]
	BTA-94299-no-rs	*MGST1*	5	9.06 × 10^−3^	BovineSNP50 Bead Chip/Illumina 54 K	Mutual	52,166	1314	Chinese Holstein	SMMA	China	[99]
	ARS-BFGL-NGS-54462	*MIR365*	25	9.06 × 10^−3^	BovineSNP50 Bead Chip/Illumina 54 K	Mutual	52,166	1314	Chinese Holstein	SMMA	China	[99]
	ARS-BFGL-NGS-102900	*AGPAT5*	27	9.06 × 10^−3^	BovineSNP50 Bead Chip/Illumina 54 K	Mutual	52,166	1314	Chinese Holstein	SMMA	China	[99]
	*apmap48553-BTA-10000*	*LOC788619*	7	9.06 × 10^−3^	BovineSNP50 Bead Chip/Illumina 54 K	Mutual	52,166	1314	Chinese Holstein	SMMA	China	[99]
	*BTB-01219012*	*LOC100296765*	7	9.06 × 10^−3^	BovineSNP50 Bead Chip/Illumina 54 K	Mutual	52,166	1314	Chinese Holstein	SMMA	China	[99]
	*ARS-BFGL-NGS-31810*	*LOC536255*	11	9.06 × 10^−3^	BovineSNP50 Bead Chip/Illumina 54 K	Mutual	52,166	1314	Chinese Holstein	SMMA	China	[99]
	rs43486059	*LOC781835*	6	3.61 × 10^−9^	GGP Bovine 100 K	No	84,407	984	Chinese Holstein	Farm CPU	China	[59]
	rs137244035	*FSTL4*	7	1.88 × 10^−8^	GGP Bovine 100 K	No	84,407	984	Chinese Holstein	Farm CPU	China	[59]
	rs43352090	*ATG4C*	3	9.91 × 10^−8^	GGP Bovine 100 K	No	84,407	984	Chinese Holstein	Farm CPU	China	[59]
	rs43366267	*SH3BP4*	3	4.11 × 10^−7^	GGP Bovine 100 K	No	84,407	984	Chinese Holstein	Farm CPU	China	[59]
PW	rs109478645	*ENSBTAG00000037537*	18	6.48 × 10^−9^	Bovine HD	HD panel	601,717	4841	Holstein	MLM	Canada	[26]
	BTB-00168895	*LOC781728*	4	9.17 × 10^−3^	BovineSNP50 Bead Chip/Illumina 54 K	Mutual	52,166	1314	Chinese Holstein	SMMA	China	[99]
	Hapmap40061-BTA-28737	*LOC616304*	9	9.17 × 10^−3^	BovineSNP50 Bead Chip/Illumina 54 K	Mutual	52,166	1314	Chinese Holstein	SMMA	China	[99]
	rs109578471	*USP6NL*	13	1.18 × 10^−7^	GGP Bovine 100 K	No	84,407	984	Chinese Holstein	Farm CPU	China	[59]
	rs42051017	*LOC101907665*	29	1.45 × 10^−7^	GGP Bovine 100 K	No	84,407	984	Chinese Holstein	Farm CPU	China	[59]
	rs43430205	*CNTN3*	22	2.24 × 10^−7^	GGP Bovine 100 K	No	84,407	984	Chinese Holstein	Farm CPU	China	[59]
RW	BTB-00752634	*LOC614209*	14	3.16 × 10^−6^	BovineSNP50 Bead Chip/Illumina 54 K	Mutual	38,720	2329	Korean Holstein	MLM	Korea	[100]
	ARS-BFGL-BAC-26802	*ANGPT1, LOC782496*	14	5.65 × 10^−5^	BovineSNP50 Bead Chip/Illumina 54 K	Mutual	38,720	2329	Korean Holstein	MLM	Korea	[100]
	ARS-BFGL-NGS-5369	*OSBP2*	17	8.45 × 10^−6^	BovineSNP50 Bead Chip/Illumina 54 K	Mutual	38,720	2329	Korean Holstein	MLM	Korea	[100]

Abbreviations: rump angle, RA; lion strength, LS; pin width, PW; rump width, RW; mixed linear model, MLM; generalized linear model, GLM; single marker mixed model analysis, SMMA; reference, Ref.

**Table 8:** GWAS study for screening genetic markers associated with mammary system traits.

Traits	SNPs	Genes	Chr	*p* Value	Genotype	Imputed	SNP Size	Sample Size	Breed	Model	Country	Ref.
Rear udder	RS-BFGL-NGS-111920	*LOC100337279*	14	8.91 × 10^−3^	BovineSNP50 Bead Chip/Illumina 54 K	Mutual	52,166	1314	Chinese Holstein	SMMA	China	[99]
	Hapmap50827-BTA-94026	*LOC100336384*	24	8.91 × 10^−3^	BovineSNP50 Bead Chip/Illumina 54 K	Mutual	52,166	1314	Chinese Holstein	SMMA	China	[99]
Udder texture	BTA-41935-no-r	*DRG1*	17	8.72 × 10^−3^	BovineSNP50 Bead Chip/Illumina 54 K	Mutual	52,166	1314	Chinese Holstein	SMMA	China	[99]
	BTB-01236227	*HTR1A*	20	8.72 × 10^−3^	BovineSNP50 Bead Chip/Illumina 54 K	Mutual	52,166	1314	Chinese Holstein	SMMA	China	[99]
	BTB-01693574	*LOC104969871*	2	1.96 × 10^−5^	BovineSNP50 Bead Chip/Illumina 54 K	Mutual	38,720	2329	Korean Holstein	MLM	Korea	[100]
	BTB-01584048	*MIR2285K-4*	26	7.57 × 10^−7^	BovineSNP50 Bead Chip/Illumina 54 K	Mutual	38,720	2329	Korean Holstein	MLM	Korea	[100]
CSL	BTB-00089278	*LRP2*	2	8.74 × 10^−3^	BovineSNP50 Bead Chip/Illumina 54 K	Mutual	52,166	1314	Chinese Holstein	SMMA	China	[99]
	BTB-01007411	*SEMA3E*	4	8.74 × 10^−3^	BovineSNP50 Bead Chip/Illumina 54 K	Mutual	52,166	1314	Chinese Holstein	SMMA	China	[99]
	ARS-BFGL-NGS-35982	*NAP1L1*	5	8.74 × 10^−3^	BovineSNP50 Bead Chip/Illumina 54 K	Mutual	52,166	1314	Chinese Holstein	SMMA	China	[99]
	ARS-BFGL-NGS-29118	*MACROD2*	13	8.74 × 10^−3^	BovineSNP50 Bead Chip/Illumina 54 K	Mutual	52,166	1314	Chinese Holstein	SMMA	China	[99]
	UA-IFASA-6670	*GABARAPL1*	5	6.37 × 10^−7^	Bovine LD V3 SNP	No	20,632	421	Chinese Holstein	MLM	China	[101]
	BovineHD0900026424	*NOX3*	9	5.03 × 10^−7^	Bovine LD V3 SNP	No	20,632	421	Chinese Holstein	MLM	China	[101]
	BovineHD1700021616	*LOC531152*	17	9.77 × 10^−7^	Bovine LD V3 SNP	No	20,632	421	Chinese Holstein	MLM	China	[101]
	BovineHD3000039710	*LOC782196*	30	5.31 × 10^−7^	Bovine LD V3 SNP	No	20,632	421	Chinese Holstein	MLM	China	[101]
	ARS-BFGL-BAC-29174	*STXBP6*	21	1.16 × 10^−9^	Bovine 100 K SNP	No	84,407	984	Chinese Holstein	Farm CPU	China	[60]
	Hapmap32447-BTC-033214	*GRID2*	6	2.45 × 10^−7^	Bovine 100 K SNP	No	84,407	984	Chinese Holstein	Farm CPU	China	[60]
	BovineHD0600005127	*LOC112447148*	6	3.02 × 10^−7^	Bovine 100 K SNP	No	84,407	984	Chinese Holstein	Farm CPU	China	[60]
Fore attachment	ARS-BFGL-NGS-114960	*NTM*	29	9.65 × 10^−3^	BovineSNP50 Bead Chip/Illumina 54 K	Mutual	52,166	1314	Chinese Holstein	SMMA	China	[99]
	ARS-BFGL-NGS-118699	*LOC511409*	8	1.96 × 10^−5^	BovineSNP50 Bead Chip/Illumina 54 K	Mutual	38,720	2329	Korean Holstein	MLM	Korea	[100]
RAW	BTB-01478363	*BAG1*	20	9.24 × 10^−3^	BovineSNP50 Bead Chip/Illumina 54 K	Mutual	52,166	1314	Chinese Holstein	SMMA	China	[99]
	Hapmap29824-BTA-137304	*SLC17A1, LRRC16A,*	23	3.76 × 10^−5^	BovineSNP50 Bead Chip/Illumina 54 K	Mutual	52,166	1314	Chinese Holstein	SMMA	China	[99]
RAH	ARS-BFGL-NGS-20052	*CDK5R2*	2	9.04 × 10^−3^	BovineSNP50 Bead Chip/Illumina 54 K	Mutual	52,166	1314	Chinese Holstein	SMMA	China	[99]
	Hapmap46979-BTA-32175	*LOC104973698*	13	9.87 × 10^−6^	BovineSNP50 Bead Chip/Illumina 54 K	Mutual	38,720	2329	Korean Holstein	GLM	Korea	[100]
	BTA-11097-rs29016861	*CDK1,RHOBTB1*	28	1.52 × 10^−5^	BovineSNP50 Bead Chip/Illumina 54 K	Mutual	38,720	2329	Korean Holstein	GLM	Korea	[100]
	rs109901274	*ARRDC3*	7	2.88 × 10^−10^	Bovine HD	HD panel	601,717	4841	Holstein	MLM	Canada	[26]
TL	rs110137797	*TMTC2*	5	1.63 × 10^−12^	Bovine HD	HD panel	601,717	4841	Holstein	MLM	Canada	[26]
	BTB-01255458	*PDIA6*	10	9.11 × 10^−3^	BovineSNP50 Bead Chip/Illumina 54 K	Mutual	52,166	1314	Chinese Holstein	SMMA	China	[99]
FTL	BovineHD1500023818	*SBF2*	15	9.69 × 10^−8^	Bovine 100 K SNP	No	84,906	984	Chinese Holstein	Farm CPU	China	[58]
	BovineHD2100009187	*STXBP6*	21	1.98 × 10^−7^	Bovine 100 K SNP	No	84,906	984	Chinese Holstein	Farm CPU	China	[58]
MGM	rs110171876	*TMTC2*	5	7.22 × 10^−8^	50 K/HD	Mutual	598,016	4578	Brown Swiss	GLM	Switzerland	[98]
	rs133549245	*RASSF6*	6	2.94 × 10^−29^	50 K/HD	Mutual	598,016	4578	Brown Swiss	GLM	Switzerland	[98]
	rs137563207	*TBX5, RBM19*	17	2.62 × 10^−46^	50 K/HD	Mutual	598,016	4578	Brown Swiss	GLM	Switzerland	[98]
	rs41584904	*PITPNA*	19	4.97 × 10^−8^	50 K/HD	Mutual	598,016	4578	Brown Swiss	GLM	Switzerland	[98]
ATP	ARS-BFGL-NGS-101241	*MMS22L*	9	5.10 × 10^−9^	Bovine 100 K SNP	No	84,906	984	Chinese Holstein	Farm CPU	China	[58]
	ARS-BFGL-NGS-43147	*E2F8*	29	4.16 × 10^−7^	Bovine 100 K SNP	No	84,906	984	Chinese Holstein	Farm CPU	China	[58]
	BovineHD1800006781	*CDH11*	18	1.09 × 10^−7^	Bovine 100 K SNP	No	84,906	984	Chinese Holstein	Farm CPU	China	[58]
	BovineHD0500031672	*PEX26*	5	2.54 × 10^−7^	Bovine 100 K SNP	No	84,906	984	Chinese Holstein	Farm CPU	China	[58]
	ARS-BFGL-NGS-16048	*TAMM41*	22	3.14 × 10^−9^	Bovine 100 K SNP	No	84,906	984	Chinese Holstein	Farm CPU	China	[58]
	ARS-BFGL-NGS-113245	*SLC39A11*	19	8.92 × 10^−3^	BovineSNP50 Bead Chip/Illumina 54 K	Mutual	52,166	1314	Chinese Holstein	SMMA	China	[99]
PTP	Hapmap58721-rs29026738	*HIVEP3*	3	3.05 × 10^−8^	Bovine 100 K SNP	No	84,906	984	Chinese Holstein	Farm CPU	China	[58]
	12-88054488-G-A-rs42352402	*MYO16*	12	6.02 × 10^−8^	Bovine 100 K SNP	No	84,906	984	Chinese Holstein	Farm CPU	China	[58]
	BTA-83107-no-rs	*MIR2284O*	6	1.10 × 10^−6^	Bovine LD V3 SNP	No	20,632	421	Chinese Holstein	MLM	China	[101]
	ARS-BFGL-NGS-31730	*SH3RF3*	11	8.64 × 10^−3^	BovineSNP50 Bead Chip/Illumina 54 K	Mutual	52,166	1314	Chinese Holstein	SMMA	China	[99]
	BTB-01230622	*DCDC5*	15	8.64 × 10^−3^	BovineSNP50 Bead Chip/Illumina 54 K	Mutual	52,166	1314	Chinese Holstein	SMMA	China	[99]
AUA	DB-340-seq-rs208014256	*MGST1*	5	4.48 × 10^−8^	Bovine 100 K SNP	No	84,407	984	Chinese Holstein	Farm CPU	China	[60]
	Hapmap58214-rs29015775	*LOC101903734*	22	8.34 × 10^−8^	Bovine 100 K SNP	No	84,407	984	Chinese Holstein	Farm CPU	China	[60]
	BovineHD2700005329	*MTUS1*	27	1.90 × 10^−7^	Bovine 100 K SNP	No	84,407	984	Chinese Holstein	Farm CPU	China	[60]
	BovineHD0900028603	*PRKN*	9	6.48 × 10^−7^	Bovine 100 K SNP	No	84,407	984	Chinese Holstein	Farm CPU	China	[60]
PUAH	BovineHD2900000083	*E2F8*	29	9.70 × 10^−8^	Bovine 100 K SNP	No	84,407	984	Chinese Holstein	Farm CPU	China	[60]
	BovineHD1800011193	*CDH11*	18	1.66 × 10^−7^	Bovine 100 K SNP	No	84,407	984	Chinese Holstein	Farm CPU	China	[60]
	BovineHD2200002408	*FOXP1*	22	4.89 × 10^−7^	Bovine 100 K SNP	No	84,407	984	Chinese Holstein	Farm CPU	China	[60]
PUAW	BovineHD0700028083	*SLF1*	7	2.26 × 10^−9^	Bovine 100 K SNP	No	84,407	984	Chinese Holstein	Farm CPU	China	[60]
	BovineHD0500010522	*TMEM117*	5	1.45 × 10^−8^	Bovine 100 K SNP	No	84,407	984	Chinese Holstein	Farm CPU	China	[60]
	BovineHD1500023322	*SBF2*	15	6.19 × 10^−8^	Bovine 100 K SNP	No	84,407	984	Chinese Holstein	Farm CPU	China	[60]
UD	BTA-75047-No-rs	*LGALS2*	5	1.26 × 10^−7^	Bovine 100 K SNP	No	84,407	984	Chinese Holstein	Farm CPU	China	[60]
	BovineHD0600024277	*GC*	6	2.92 × 10^−7^	Bovine 100 K SNP	No	84,407	984	Chinese Holstein	Farm CPU	China	[60]
	BovineHD0600001885	*UBE2K*	6	5.16 × 10^−7^	Bovine 100 K SNP	No	84,407	984	Chinese Holstein	Farm CPU	China	[60]
	BovineHD0900001933	*ADGRB3*	9	5.98 × 10^−7^	Bovine 100 K SNP	No	84,407	984	Chinese Holstein	Farm CPU	China	[60]
	BovineHD2300001734	*GCLC*	23	9.36 × 10^−7^	Bovine 100 K SNP	No	84,407	984	Chinese Holstein	Farm CPU	China	[60]

Abbreviations: central suspensory ligament, CSL; rear attach width, RAW; rear attach height, RAH; teat length, TL; fore teat length, FTL; mammary gland morphology, MGM; anterior teat position, ATP; posterior teat position, PTP; anterior udder attachment, AUA; posterior udder attach height, PUAH; posterior udder attach width, PUAW; udder depth, UD; mixed linear model, MLM; generalized linear model, GLM; single marker mixed model analysis, SMMA; reference, Ref.

## Data Availability

Not applicable.

## Supplementary Materials

The following supporting information can be downloaded at: https://www.mdpi.com/article/10.3390/ani14152181/s1, Table S1: The relationship between body linear type traits and dairy cow production, reproduction and health performance.
