# Supplementary material for: Genome-Wide Association Study as an Efficacious Approach to Discover Candidate Genes Associated with Body Linear Type Traits in Dairy Cattle
Source: Animals (Basel). 2024 Jul 26;14(15):2181. doi: 10.3390/ani14152181 (PMC11311069; doi:10.3390/ani14152181)
Supplement: Supplementary file 1 [file animals-14-02181-s001.zip › animals-3079590-supplementary.pdf]

Table S1. The relationship between body linear type traits and dairy cow production, reproduction and health performance

Traits	RFI [18]		LAEI [18]		Milk yield [19] [28]		Body weight [19]		Longevity [23]		Calving interval [38]		Healthy traits [37]	
	R_g	R_p	R_g	R_p	R_g	R_p	R_g	R_p	R_g	R_p	R_g	R_p	R_g	R_p
Stature	0.350	0.120	0.470	0.290	-0.484	0.211	0.815	0.547	-0.210	-0.020	0.330	NR	NR	NR
Chest width	0.390	0.170	0.550	0.330	0.349	0.178	0.400	0.480	-0.200	-0.020	0.280	NR	NR	NR
Body depth	0.110	0.140	0.340	0.210	-0.257	0.274	0.205	0.647	-0.280	-0.050	0.260	NR	NR	NR
Body height	0.110	0.060	0.120	0.050	0.594	0.236	0.822	0.379	NR	NR	NR	NR	NR	NR
Body length	NR	NR	NR	NR	0.048	0.250	0.179	0.297	-0.090	-0.010	NR	NR	NR	NR
Pin width	0.460	0.150	0.330	0.210	0.088	0.109	0.180	0.348	-0.070	-0.020	-0.020	NR	-0.110[c]	-0.030[c]
Rear udder height	0.110	0.110	0.080	-0.060	-0.300	0.034	0.844	0.353	0.200	0.020	0.230	NR	NR	NR
Rear udder width	0.450	0.170	0.520	0.390	0.619	0.147	0.621	0.294	-0.160	-0.010	0.190	NR	NR	NR
Rear teat placement	NR	NR	NR	NR	-0.465	-0.140	-0.038	-0.149	0.170	-0.010	-0.010	NR	NR	NR
Fore teat position	NR	NR	NR	NR	0.762	0.082	-0.139	-0.249	0.100	-0.010	0.440	NR	0.070[a]	0.020[a]
Teat length	NR	NR	NR	NR	0.405	0.141	-0.881	-0.227	0.110	0.000	0.090	NR	0.370	0.030[a]
Central suspensory ligament	NR	NR	NR	NR	-0.184	0.159	0.019	0.220	0.030	0.020	0.210	NR	NR	NR
Udder depth	0.140	0.040	0.090	-0.150	-0.129	-0.290	0.373	0.405	0.310	0.070	NR	NR	0.000[a]	0.000[a]
Feet and legs	−0.340	−0.110	−0.060	0.050	NR	NR	NR	NR	NR	NR	NR	NR	0.090[b]	-0.040[b]
Bone quality	NR	NR	NR	NR	NR	NR	NR	NR	0.230	0.020	NR	NR	-0.060[b]	-0.060[b]
Foot angle	0.410	0.120	0.370	0.240	-0.060	-0.080	NR	NR	-0.170	0.000	-0.200	NR	-0.360[b]	-0.020[b]
Rear leg side view	NR	NR	NR	NR	0.060	0.060	NR	NR	-0.010	0.000	NR	NR	NR	NR
Rear leg rear view	NR	NR	NR	NR	0.040	0.010	NR	NR	NR	NR	0.190	NR	0.030[b]	0.370[b]

Abbreviations: residual feed intake, RFI; actual energy intake, LAEI; the value of genetic correlation, R_g; the value of phenotype correlation, R_p; Mastitis, a; Lameness, b; Retained placenta, c; no record founded, NR,
